# Serum uric acid levels and cancer mortality risk among males in a large general population-based cohort study

**DOI:** 10.1007/s10552-014-0408-0

**Published:** 2014-06-07

**Authors:** N. Taghizadeh, J. M. Vonk, H. M. Boezen

**Affiliations:** 1 Department of Epidemiology, University of Groningen, University Medical Center Groningen, FA40, E3-29, Hanzeplein 1, 9700 RB Groningen, The Netherlands; 2 GRIAC Research Institute, University of Groningen, University Medical Center Groningen, Groningen, The Netherlands

**Keywords:** Cancer mortality, Serum uric acid, Cholesterol, Triglyceride

## Abstract

**Purpose:**

Serum uric acid (SUA) has antioxidant capacities and therefore may protect against the development of cancer. Few epidemiological studies have tested this hypothesis, and findings were inconsistent.

**Methods:**

We studied the association between SUA levels and mortality due to any type of cancer, and three common types of cancer among males (lung, colorectal, and prostate cancer) in the general population-based Vlagtwedde–Vlaardingen cohort with 38 years of follow-up and 8 surveys (total number of males = 4,350). Of 1,823 males with data available on SUA, 254 (13.9 %) died due to any cancer (lung *n* = 75 (4.1 %), colorectal *n* = 27 (1.5 %), and prostate cancer *n* = 23 (1.3 %), assessed on 31 December 2008). SUA, cholesterol, and triglyceride were measured in males during the surveys in 1970, 1972, and 1973. We analyzed the association between cancer mortality risk and SUA level both as continuous variable and as tertiles: lowest <5 mg/dl (reference), middle 5–5.8 mg/dl, and highest >5.8 mg/dl, using multivariate Cox regression with adjustment for age, smoking (pack years), and body mass index.

**Results:**

Higher levels of SUA were associated with a lower risk of mortality from any cancer [HR (95 % CI) = 0.85 (0.73–0.97)]. SUA levels in the highest tertile (>5.8 mg/dl) were associated with a lower risk of mortality from any cancer [0.68 (0.48–0.97)]. Additional adjustment for serum total cholesterol and triglyceride levels did not change the results.

**Conclusions:**

Our study indicates that elevated SUA levels may protect against cancer mortality.

## Introduction

Several studies have linked serum uric acid (SUA) levels to general mortality, cardiovascular disease, or the metabolic syndrome [1, 2], but only a few epidemiological studies have addressed the association between SUA and cancer mortality [3–7]. Moreover, the results of these few studies are inconsistent. SUA is derived from the oxidation of xanthine and hypoxanthine (products of purine nucleotides) by the enzyme xanthine oxidoreductase (XOR) [8, 9]. In humans, normal SUA level is between 3 and 6.8 mg/dl. A high SUA level can be derived from purine (purine nucleotides, adenine, and guanine)-rich diets (e.g., proteins), renal dysfunction in excretion of SUA [9], or as a result of increased cell apoptosis and necrosis [4]. SUA as an antioxidant was hypothesized to protect against cancer development by its capacity to scavenge oxygen radicals and inhibit lipid peroxidation [10]. However, results from other studies do not support this hypothesis, suggesting a pro-inflammatory role of SUA in specific diseases [4, 9, 11].

The role of SUA as independent factor in the development of cancer is controversial. Evidence shows there are positive associations between SUA and established risk factors for cardiovascular disease, metabolic syndrome, and cancer such as body mass index (BMI), levels of cholesterol, and triglyceride [4, 5]. Thus, several confounding factors underlying these comorbidity disorders may have been responsible for a potential link between SUA levels and cancer.

We aimed to study the association between SUA levels and cancer mortality and investigate whether this association is independent of an individual’s cholesterol and triglyceride levels. The current study examines the association between SUA and mortality due to any type of cancer, and due to the three most common types of cancer in males, i.e., lung, colorectal, and prostate cancer, using the Vlagtwedde–Vlaardingen cohort [12, 13]. This large cohort has been followed up for 38 years and offers us the unique possibility to investigate this association.

## Methods

### Study population

We studied the association between SUA and mortality from cancer using the Vlagtwedde–Vlaardingen cohort study. This study has been described in detail previously [12, 13]. In brief, the Vlagtwedde–Vlaardingen study was set up as a general population-based cohort study on the epidemiology of pulmonary diseases in exclusively Caucasian individuals of Dutch descent. The study started in 1965, and participants had medical examinations every 3 years until the last survey in 1989/1990. In Vlaardingen, only participants who were included at baseline (1965 or 1969) were approached for follow-up, whereas in Vlagtwedde new subjects aged between 20 and 65 years were invited to participate at every survey. The number of surveys per subject ranges from 1 to 8 (median number of surveys per subject: two). The final surveys were organized in 1989 in Vlagtwedde and in 1990 in Vlaardingen. SUA, cholesterol, and triglyceride were measured in males only during the surveys in 1972 (measured in Vlaardingen), and 1970 and 1973 (measured in Vlagtwedde). Blood sampling was performed in a fasting condition. We updated the vital status of all participants in the Vlagtwedde–Vlaardingen study on 31 December 2008 and evaluated mortality outcomes, i.e., any cancer mortality, and three common types of cancer mortality, being lung cancer, colorectal cancer, and prostate cancer, either as primary or secondary cause of death. Analyses on cause-specific mortality were performed at Statistics Netherlands (The Hague, The Netherlands).

### Ethics statement

The committee on human subjects in research of the University of Groningen reviewed the study and affirmed the safety of the protocol and study design and specifically approved this study. All participants gave their written informed consent.

### Population characteristics

We collected data on age, sex, smoking habits, and place of residence using the Dutch version of the British Medical Research Council questionnaire [12].

### Serum uric acid, serum total cholesterol, and triglyceride

Fasting SUA level was determined according to the EDTA-hydrazine method on an autoanalyzer [14]. Fasting serum total cholesterol was determined according to the Huang method [15] on an autoanalyzer. Fasting serum triglycerides concentration was determined according to the Laurell method [16].

We included only SUA, cholesterol, and triglyceride level of a male’s first available measurement at survey 1970, 1972, or 1973.

We analyzed SUA levels as a continuous variable and as tertiles:

1. SUA <5 mg/dl (as reference);
2. SUA 5–5.8 mg/dl
3. SUA >5.8 mg/dl

### Cancer mortality

Cancer mortality was classified according to the international classification of diseases (ICD) coding system: any cancer (ICD 7: 140–239, 294; ICD 8: 140–239; ICD 9: 140–239 and 288; ICD 10: C00–C97, D00–D48); lung cancer (cancer of trachea, bronchus, and lung) (ICD 7: 162, 163; ICD 8: 162, 163; ICD 9: 162, 163, 165; and ICD 10: C33, C34, C38, and C39); colon and rectal cancer (colorectal cancer) (ICD 7: 153, 154; ICD 8: 153, 154; ICD 9: 153, 154; ICD: 10 C18–C21); and prostate cancer (ICD 7: 177; ICD 8: 185; ICD 9: 185 and ICD: 10: C61).

### Statistical analyses

First, descriptive analyses of the male characteristics and the mortality statistics were performed. Independent sample *t* test, Mann–Whitney *U* test, and chi-square test were used to determine significant differences between groups for continuous and categorical variables, respectively. Triglyceride levels were log-transformed to obtain normality of the distribution. Secondly, hazard ratios associated with SUA levels for mortality from any cancer and any of the three common types of cancer were estimated using multivariate Cox regression. The Cox regression model was adjusted for age, BMI, and smoking (pack years), all at first available survey with data on SUA. Additionally, we adjusted the Cox regression model for cholesterol and triglyceride levels. In the Cox regression analyses, censoring took place when the males were still alive, were lost to follow-up, or died of causes other than cancer or the specific cancer under study [17]. Time was defined from the first available survey with data on SUA, until mortality from any type of cancer or specific cancer as the end point of interest or until censoring. *p* values <0.05 (tested two-sided) were considered to be statistically significant.

## Results

Of all 4,350 males, 2,249 were examined in 1970, 1972, or 1973. In total, 1,823 (81 %) males had data available on SUA. Subjects who had data available on SUA had a higher BMI level and a higher number of pack years compared to subjects who were examined in 1970, 1972, or 1973 but had no data available on SUA. Vital status of subjects who had data available on SUA was also significantly different compared to those with no data on SUA (results not shown). Of those males with data available on uric acid, 1,162 (63.7 %) were alive, 254 (13.9 %) died due to any cancer, 354 (19.4 %) died but not due to cancer, and 29 (1.6 %) died due to external causes (e.g., an accident, suicide, or homicide), in 5 (0.3 %) males the cause of death could not be determined and 19 (1.0 %) were lost to follow-up (Table 1). Of those males who died due to cancer, 75 (29.5 %) died due to lung cancer, 27 (10.6 %) died due to colorectal cancer, and 23 (9.1 %) died due to prostate cancer (Table 1). The mean age at the first SUA survey of males who died due to cancer was 42.3 (SD = 8.9). Males who died due to cancer had a significantly lower level of SUA compared to males who were alive.

**Table 1:** Vital status of males at their first available survey with data available on SUA in the general population of Vlagtwedde–Vlaardingen (*n* = 1,823)

Characteristics^a^	Alive (*n* = 1,162)	Died due to any cancer (*n* = 254)	Died, but not due to cancer (*n* = 354)	Died due to external causes (*n* = 29)	Lost to follow-up (*n* = 19)
Age (years), mean (SD)	31.9 (8.5)	42.3 (8.9)^b^	44.2 (8.0)	38.3 (11.3)	30.7 (9.7)
Smoking (pack years), median (range)	6.0 (0.0–82.2)	16.4 (0.0–80.4)^b^	18.4 (0.0–68.2)	15.0 (0.0–78.7)	5.5 (0.0–31.5)
BMI kg/m^2^, mean (SD)	25.0 (3.0)	25.5 (3.0)^b^	25.9 (3.0)^c^	25.9 (3.5)	25.2 (2.5)
SUA (mg/dl), mean (SD)	5.5 (0.9)	5.2 (1.0)^b^	5.4 (1.0)	5.5 (1.2)	5.8 (1.1)
Tertiles of SUA (mg/dl), *n* %					
<5	369 (31.7)	108 (42.5)^b^	135 (37.8)	12 (40.0)	4 (21.1)
5–5.8	423 (36.3)	88 (34.6)	120 (33.6)	8 (26.7)	6 (31.6)
>5.8	372 (32.0)	58 (22.8)	103 (28.8)	10 (33.3)	9 (47.4)
Serum total cholesterol (mg/dl), mean (SD)	235.8 (45.6)	253.9 (42.5)^b^	261.3 (48.3)^c^	251.0 (47.1)	244.2 (44.0)
Triglyceride (mg/dl) (Log 10), mean (SD)	2.0 (0.2)	2.0 (0.2)	2.1 (0.2)^c^	2.0 (0.3)	2.0 (0.2)

^a^Males: *n* = 1,823; in 5 (0.3 %) males, the cause of death could not be determined

^b^Significantly different from males who were alive (*p* value calculated by chi-square or *t* test <0.05)

^c^Significantly different from males who died due to any type of cancer (*p* value calculated by chi-square or *t* test <0.05)

### Serum uric acid

**Fig. 1:**
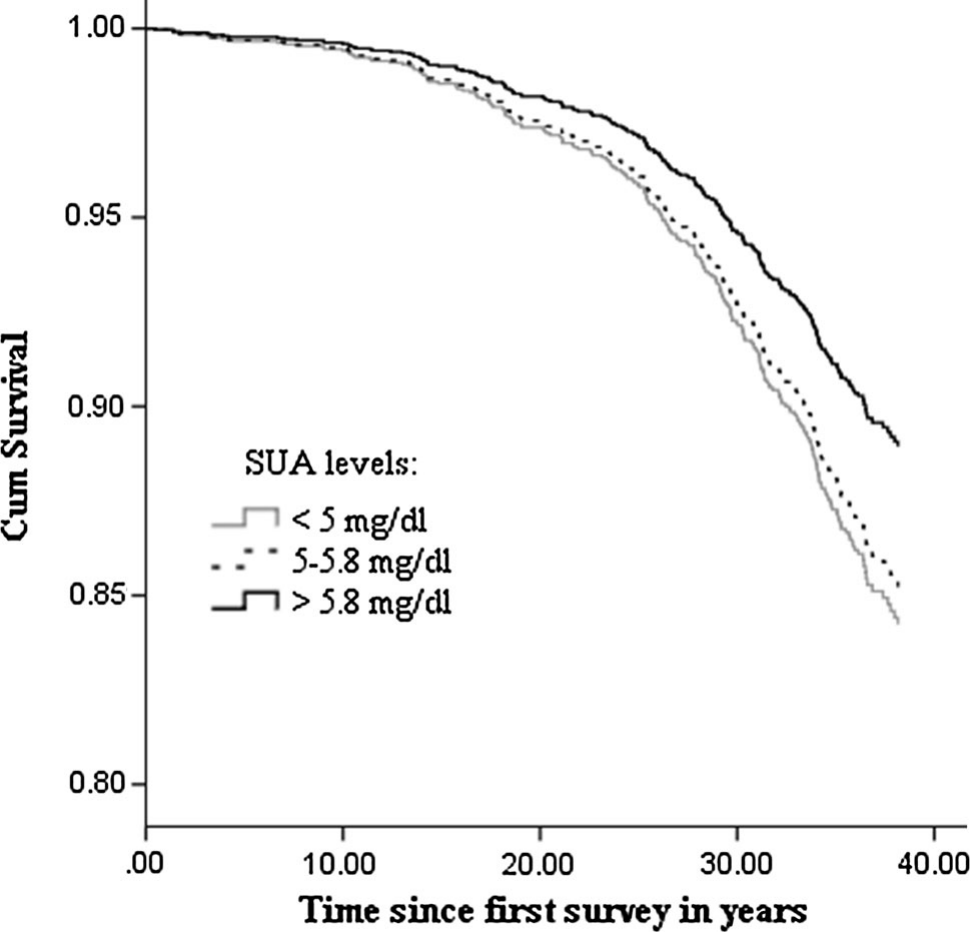
Cox proportional hazard survival curves according to different categories of SUA at first available survey on any types of cancer mortality risk for males

Higher levels of SUA were significantly associated with a lower risk of mortality from any cancer [HR (95 % CI): 0.85 (0.73–0.97)] (Table 2-model 1). SUA levels higher than 5.8 mg/dl were significantly associated with a lower risk of mortality from any cancer [0.68 (0.48–0.97)] (Table 2-model 1; Fig. 1). Additional adjustment for total blood cholesterol and triglyceride level did not change the results (Table 2-model 2).

**Table 2:** Hazard ratio (with 95 % confidence interval) of serum uric acid (SUA) for mortality from any cancer, lung cancer, colorectal cancer, and prostate cancer among 1,823 males *(n* = 1,723 without missing values on confounders). Cox regression models with adjustment for age, BMI, smoking (pack year) at the first survey with data available on SUA. Additional adjustments for lipid profile (cholesterol and triglyceride) in model 2 (*n* = 1,713 without missing values on confounders)

	Any cancer				Lung cancer			Colorectal cancer			Prostate cancer	
	N events	HR	95 % CI	N events	HR	95 % CI	N events	HR	95 % CI	N events	HR	95 % CI
*Model 1: Without adjustment for lipid profile*												
SUA mg/dl	235	**0.85**	**0.73–0.97**	75	0.77	0.60–1.00	24	1.02	0.65–1.58	18	0.62	0.37–1.04
SUA tertiles, mg/dl												
<5.0	103	1		34	1		10	1		9	1	
5.0–5.8	82	0.93	0.70–1.25	28	0.97	0.59–1.60	9	1.27	0.51–3.19	8	1.19	0.46–3.17
>5.8	50	**0.68**	**0.48–0.97**	13	0.53	0.27–1.01	5	0.84	0.28–2.50	1	0.14	0.02–1.12
*Model 2: With adjustment for lipid profile*												
SUA mg/dl	234	**0.86**	**0.75–1.00**	74	0.84	0.64–1.08	24	1.04	0.66–1.65	18	0.60	0.35–1.01
SUA tertiles, mg/dl												
<5.0	102	1		33	1		10	1		9	1	
5.0–5.8	82	0.95	0.70–1.27	28	1.01	0.60–1.69	9	1.31	0.51–3.33	8	1.13	0.43–3.00
>5.8	50	**0.70**	**0.49–0.99**	13	0.55	0.28–1.08	5	0.88	0.29–2.69	1	0.12	0.01–1.02

Statistically significant results are shown in bold

## Discussion

This is the first study showing that SUA levels are associated with a lower risk of mortality from any type of cancer in males from a general population cohort followed up for 38 years. This association remained after adjustment for serum total cholesterol and triglyceride levels.

Ames et al. [10] for the first time hypothesized that SUA provides a primary defense against human cancer by its role as a scavenger of singlet oxygen, hydroxyl radical (a product of singlet oxygen conversion), and suppresses the lipid peroxidation in erythrocytes. Further support for the antioxidant role of SUA has been identified in many different organ systems [18].

While there is some support for this hypothesis among epidemiological studies, this idea has been strongly supported by molecular investigations, suggesting that an indirect pharmacological reduction in SUA levels, by inhibiting the XOR receptor, provokes tumorigenesis [19, 20]. Another example that supports the antioxidant capacity of SUA is a recent study on the link between a broad range of oxidative stress parameters and colon cancer survival [21]. Authors reported that among plasma antioxidants only higher uric acid levels were associated with longer survival among colon cancer patients [21]. They suggested that SUA acts as the main antioxidant by scavenging the free radicals and stabilizing ascorbic acid in human serum [21, 22]. However, this study was performed among cancer patients. So far, there is no evidence to show whether SUA levels in healthy males provide a primary defense against cancer.

The majority of studies on the potential antioxidant properties of SUA are based on mortality in general, so reliable information about subgroup and different cancer types is scarce. Moreover, only few studies have controlled for the blood lipid profile of the subjects [4, 6]. One of these studies with control for lipid profile found no association between SUA levels and cancer mortality after adjustment for age, current smoking, diabetes, hypertension, and hypercholesterolemia [4]. However, this study was restricted to a specific population; the SUA measurements were obtained only among subjects who had an abnormal value of total cholesterol or urinary protein levels [4].

Others have reported that high levels of SUA were associated with a higher risk of mortality from cancer among males after adjustment for established risk factors such as cholesterol [6, 7]. The finding of an increased risk of cancer can be explained by the fact that SUA levels may have been altered by preexisting disease (cancer) at the time of measurement since hyperuricemia can be an indicator of the tumor lysis syndrome. The tumor lysis syndrome is a result of the rapid intracellular release of dying cancer cell’s contents, which may lead to hyperuricemia as well as other metabolic disorders [23]. Thus, an increased nucleic acid turnover in the rapid proliferation of neoplastic cells may lead to an increased level of SUA [7]. A sensitivity analysis, excluding those subjects who died within 5 years of the baseline SUA measurement (*n* = 26 of which eight died of cancer), resulted in similar associations between SUA level and cancer mortality, indicating that the effect of possible inclusion of cancer patients at baseline is minimal in our study (results not shown).

It is also possible that the increased risk to develop cancer may not be due to SUA, but that high SUA levels rather reflect an unhealthy lifestyle with an increased cancer risk [6].

The association between SUA levels and cancer is known to vary by cancer type. One study reported that SUA levels were positively associated only with cancer mortality of digestive and respiratory systems, and intra-thoracic organs [6]. However, we did not find this association.

Based on the concentrations, some studies reported a U-shaped or J-shaped association between SUA and cancer mortality [3, 24, 25]. Kua et al. [3] reported a U-shaped association between SUA and all-cause and cancer mortality, with a negative association between SUA levels and mortality when the SUA levels range from 0.3 to 0.4 mmol/l, which is equivalent to 5–6.9 mg/dl The vast majority of our population (59.1 %) had SUA levels between 5 and 6.9 mg/dl; thus, our negative association is in accordance with the results of Kua et al. [3]. The mean SUA level in our population is lower than reported in other studies (mean = 5.4 mg/dl). This can be explained by the fact that in our study SUA levels were measured between 1970 and 1973 while other studies were more recent. In recent decades, SUA levels in the general population tend to increase, which is due to various changes in lifestyle factors including increased purine-rich diets and alcohol consumption [26]. Thus, having a lower range of SUA levels in our population compared to other general populations seems to be reasonable.

However, data from epidemiological studies suggest that the beneficial or detrimental effects of SUA are not only dependent on its concentration, but also influenced by several metabolic environmental and genetic factors [27], which makes comparisons across studies difficult.

Although information on major risk factors such as age, BMI, and cholesterol and triglyceride was collected, our study did not assess some other factors that may affect SUA level such as impaired renal function, hypertension, diabetes, and diet. Another limitation is that the SUA measurements were available only in males and thus results cannot be generalized to females. Finally, the impact of changes in SUA levels during follow-up on risk of cancer mortality could not be investigated in our study. Therefore, the risk of cancer mortality in relation to SUA could be underestimated by considering only single measurements (i.e., at baseline). Thus, further studies are needed to clarify the association between SUA and risk of cancer mortality.

The major strength of our current study is the longitudinal design. We were able to follow our participants for 38 years, which provided a unique wide time window for evaluating the risk of SUA levels on cancer mortality. Other strengths of our study are the large number of males included, and the high follow-up rate, since 98.5 % of the included males could be traced back [28].

In conclusion, this study shows that a higher SUA level is associated with a lower risk of cancer mortality.

## Abbreviations

SUA: Serum uric acid
HR: Hazard ratio
ICD: International classification of diseases
BMI: Body mass index
Mg/dl: Milligram/deciliter
